# The Grasp Reflex and Moro Reflex in Infants: Hierarchy of Primitive Reflex Responses

**DOI:** 10.1155/2012/191562

**Published:** 2012-06-11

**Authors:** Yasuyuki Futagi, Yasuhisa Toribe, Yasuhiro Suzuki

**Affiliations:** Department of Pediatric Neurology, Osaka Medical Center and Research Institute for Maternal and Child Health, 840 Murodo-cho, Izumi, Osaka 594-1101, Japan

## Abstract

The plantar grasp reflex is of great clinical significance, especially in terms of the detection of spasticity. The palmar grasp reflex also has diagnostic significance. This grasp reflex of the hands and feet is mediated by a spinal reflex mechanism, which appears to be under the regulatory control of nonprimary motor areas through the spinal interneurons. This reflex in human infants can be regarded as a rudiment of phylogenetic function. The absence of the Moro reflex during the neonatal period and early infancy is highly diagnostic, indicating a variety of compromised conditions. The center of the reflex is probably in the lower region of the pons to the medulla. The phylogenetic meaning of the reflex remains unclear. However, the hierarchical interrelation among these primitive reflexes seems to be essential for the arboreal life of monkey newborns, and the possible role of the Moro reflex in these newborns was discussed in relation to the interrelationship.

## 1. Introduction

Both the palmar grasp reflex and the plantar grasp reflex are very primitive in the sense that they can be elicited in all normal preterm infants at as early as 25 weeks of postconceptional age (PCA) [1]. During routine ultrasound examination, fetal palmar reflex grasping of the umbilical cord has been repeatedly observed, which first appears at 16 weeks' gestation [2–4]. These grasp reflexes are easy to elicit but have been proved to be of distinctive clinical significance for the early detection of infants with neurodevelopmental abnormalities [5–8].

 The Moro reflex was first described by Ernst Moro in 1918 [9]. This reflex is also primitive, being seen in some preterm infants at 25 weeks of PCA and in the majority by 30 weeks of PCA [1]. Since his report, many authors have devised a variety of methods for eliciting the reflex, and the underlying neural mechanism including afferent pathways of the reflex has been a subject of considerable discussion [10–13]. The phylogenetic meaning of this reflex also remains unclear [9, 10].

 It is interesting that, in spite of the great difference in the motor behavior, there is a close interrelationship between these primitive reflexes in the responses: the palmar grasp reflex inhibits the Moro reflex [12, 14–17]. This paper mainly concerns the clinical significance and neural mechanism of the grasp reflex and the Moro reflex and also attempts to discuss the meaning of these reflexes based on comparison of the responses in human infants with those in monkey infants and the hierarchical interrelation of the responses of the primitive reflexes.

## 2. The Grasp Reflex

### 2.1. Elicitation

To elicit the palmar grasp reflex, the examiner inserts his or her index finger into the palm of the infant from the ulnar side and applies light pressure to the palm, with the infant lying on a flat surface in the symmetrical supine position while awake [18–20]. Tactile without pressure and nociceptive stimulation of the palm are both inadequate. The response of the reflex comprises flexion of all fingers around the examiner's finger, which is composed of two phases: finger closure and clinging. The latter occurs as a reaction to the proprioceptive stimulation of the tendons of the finger muscles due to slight traction subsequent to the application of pressure to the palm [21, 22].

 The plantar grasp reflex is elicited by pressing a thumb against the sole of a foot just behind the toes [6, 18, 23]. The state and position are the same as for eliciting the palmar grasp reflex. The response of the reflex consists of flexion and adduction of all the toes. Milani-Comparetti and Gidoni [24] devised another method for eliciting the plantar grasp reflex. They tested for the presence of the reflex by placing the infant in a supported standing position, stimulating the soles of the feet by floor contact, looking for plantar flexion of the toes. Determination of whether the response of the hands and feet is a true reflex or a voluntary grasping movement can be difficult in older infants. An examiner should test several times with an appropriate interval between the tests, carefully observing the infant's behavior [8, 25].

### 2.2. Clinical Significance

#### 2.2.1. Responses in Normal Infants

The grasp reflexes of the hands and feet in normal term infants have been studied by several authors. Their results were fairly consistent regarding the times of the appearance and disappearance of the reflexes. The palmar grasp reflex and the plantar grasp reflex can be elicited in all infants during the first 3 and 6 months of age, respectively. Thereafter they decrease along with the intensity of the responses, usually disappearing by 6 and 12 months of age, respectively [6, 7, 25–27]. The disappearance of the reflexes is significantly related to the commencement of the voluntary use of hands or standing [28, 29].

 In contrast to the studies involving term infants, those involving preterm infants have been few. Allen and Capute [1] examined 47 infants and concluded that the intensity of the primitive reflexes including the grasp reflexes of the hands and feet in the preterm infant at term (40 weeks of PCA) was similar to that in full-term newborns reported in the literature. We analyzed the data for 834 infants (332 term and 502 preterm) who were later confirmed to be normal on follow-up examination between ages 3 to 9 years [6]. The primitive reflex responses of preterm infants were compared with those of term infants, according to corrected age as to expected birth date. No difference was evident in the changes in responses including that of the plantar grasp reflex between term and preterm infant groups throughout the first year of life.

#### 2.2.2. Abnormal Responses

In general, a primitive reflex in infants is regarded as abnormal when it is absent or diminished during the period it should be actively elicitable or lasts beyond the normal age limit for its disappearance. An exaggerated reflex can also be abnormal. The response of the palmar grasp reflex may be less intense during the first and second days after birth [18]. The absence of this reflex usually reflects peripheral (i.e., root, plexus, or nerve) or spinal cord involvement, especially regarding asymmetrical responses [18, 30, 31]. However, lesions of the upper brain structures also can affect the response. The response may be increased and retained longer, compared with that in normal infants, on the affected side(s) of the upper limb(s) in infants with spastic hemiplegia or quadriplegia, whereas it is very weak in infants with cerebral palsy (CP) of the athetoid type [26, 27, 32].

The clinical value of the plantar grasp reflex in infants has been investigated in more detail than that of the palmar grasp reflex. In 1932, Brain and Curran [33] reported two cases showing no response at the age of 6 months who both suffered from bilateral marked spasticity and one case showing a vigorous response at the age of 2.5 years who suffered from congenital choreoathetosis. They also investigated the reflex in 59 patients with Down's syndrome, ranging in age from 1 to 45 years. They discovered that up to age 20 years, the response was present in 25 of 42 patients and, after age 20 years, in 3 of 17. We analyzed 617 infants (291 term and 326 preterm) whose plantar grasp reflex was examined before 1 year of age [5]. Their diagnoses were confirmed by follow-up examinations and comprised normality in 458, CP in 78, and mental retardation (MR) in 81. We obtained several results: the reflex reactivity in infants with CP of the spastic type was significantly reduced; infants with CP of the athetoid type exhibited an extremely strong retention of the reflex; infants with MR also exhibited a tendency for prolonged retention of the reflex; the reflex profile in infants with CP of the athetoid type with spasticity, or of the ataxic type, was not different from that in normal control subjects. Other authors also reported the characteristics of the plantar grasp reflex in children with neurodevelopmental abnormalities of various types, and their results were well consistent with ours [26, 27, 34].

 A reduced or negative plantar grasp reflex during early infancy can be a sensitive indicator of later development of spasticity. Of 2267 infants whose plantar grasp reflex had been examined before 1 year of age, we analyzed the neurodevelopmental outcome in 47 infants exhibiting a negative response during the first 6 months of age and in 46 infants exhibiting a significantly reduced response at ages 1 to 4 months [6]. The diagnoses comprised CP in 75, MR in 7, borderline intelligence in 2, motor delay in 1, and normality in 8. Of the 75 cases with CP, 69 had the spastic type and 4 the athetoid type with spasticity. The total number of patients with CP of the spastic type or the mixed type with spasticity among the entire 2267 infants in this series was 107, and 73 of these 107 patients with CP with spasticity (68.2%) exhibited a negative or reduced response during early infancy. In the remaining 34 infants with CP with spasticity who exhibited a normal response during the corresponding period, the extent of motor disturbance was significantly milder [35].

 A high concordance between the side of an abnormal plantar grasp reflex during infancy and the laterality of the disturbance of motor function in children with CP of the spastic type was demonstrated [36]. The side affected, or more affected, in motor function was in accordance with the side that exhibited a diminished or more diminished response in most subjects with spastic hemiplegia, diplegia, and quadriplegia.

### 2.3. Neural Mechanism

#### 2.3.1. Spinal Reflex Center and Higher Brain Mechanism

Because anencephalic infants demonstrate a positive grasp reflex in both the hands and feet, the cerebral hemispheres are apparently not necessary for the reflexes [37–39]. Shahani et al. [40] reported that the latency of the palmar grasp reflex was 40 msec on direct electrical stimulation of the afferent fibers in the median and ulnar nerves at the level of the wrist in an adult patient who showed the palmar grasp reflex following a vascular lesion in the cerebral hemisphere. This short latency excludes the long cerebral reflex arcs and puts this grasp reflex in the category of segmental reflexes mediated at the level of the spinal cord.

 The spinal reflex center, however, is controlled by a higher brain mechanism. The grasp reflexes can be elicited in neonates and early infants as a result of insufficient control of the spinal mechanism by the immature brain, but the reflexes gradually disappear with age, due to the increased inhibition accompanying brain maturation [23, 41]. Adult patients with lesions in the frontal lobes sometimes exhibit a grasp reflex of the hands and feet [23, 33, 37, 41–45]. Such reappearance of these reflexes in adults is attributed to the release of the spinal reflex center from the disturbed higher brain mechanism, suggesting that these reflexes are only inhibited, and not lost, after the infantile period.

 Many attempts have been made to determine the pathological site of the grasp reflexes by means of clinical observation or animal experiments [37, 46–48]. In 1927, Adie and Critchley [37] analyzed 13 patients with a tumor or vascular lesion in a frontal lobe who exhibited a palmar grasp reflex on the side opposite to that of the diseased frontal lobe. They confirmed that the palmar grasp reflex was most evident when pyramidal signs were absent, and thus they concluded that the lesion responsible for the reflex was extrapyramidal. In 1938, Goldstein [41] described that a lesion involving the medial aspect of a frontal lobe seemed to be especially prone to produce the plantar grasp reflex on the opposite side. More recent studies have implicated lesions of the medial or lateral frontal cortex anterior to the primary motor area, that is, the supplementary motor area (SMA), premotor cortex, and cingulate motor cortex, as the etiology of the palmar grasp reflex [23, 45, 48, 49]. These findings indicate that nonprimary motor areas comprise substantial portions of the brain that exert inhibitory control over the spinal reflex mechanism underlying the grasp reflexes and that the destruction of these structures will release the inhibitory control and thus lead to the reappearance of the reflexes.

#### 2.3.2. Descending Control of the Spinal Circuit by the Nonprimary Motor Cortex

Nonprimary motor areas play important roles in the planning, preparation, initiation, and execution of motor behavior [50–55]. These areas contain corticospinal neurons and thereby a somatotopically arranged map of the body [54, 56–58]. However, the cortical projections from these areas to the spinal cord do not necessarily have a direct influence on spinal motor neurons. The majority of the neurons terminate in the intermediate zone in the spinal cord and connect with interneurons [53, 56, 59, 60]. The essential roles of nonprimary motor areas in the spinal cord are thought to be in the preparation and modulation of the spinal circuitry through interneurons rather than the generation of movements that require a direct command to the spinal motor neurons [52, 53, 60, 61]. On the other hand, because interneurons are substantially related to the modulation of spinal reflexes [52, 57], the descending inhibitory control of the grasp reflexes by nonprimary motor areas may also occur through spinal interneurons. As brain maturation proceeds, increasing control of the spinal circuit by the nonprimary motor areas will replace the simple reflex grasping during early infancy, with more regulatory and adaptive voluntary grasping in older infants.

 However, the proportion of patients with a lesion of a nonprimary motor area in whom a grasp reflex can be elicited is not necessarily high. In the series of De Renzi and Barbieri [44], the palmar grasp reflex was elicited in 21 of 32 (66%) patients with a medial frontal lesion and in 8 of 30 (26%) with a lateral frontal lesion. On the other hand, a minority of patients with a deep lesion including the basal ganglia without frontal cortical damage were reported to exhibit a positive palmar grasp reflex [44], and the extension of an SMA lesion into more lateral regions of area 6 may increase the strength of the grasp reflex [48]. These observations seem to indicate that the inhibitory stimuli from upper brain structures travel via multiple routes [44], and the extent of the release of control depends not only on the specificity of the location but also on the extent and severity of the lesions.

 Clinical studies have revealed that some patients with a frontal lesion exhibit a grasp reflex of the hands or feet, or both [33, 41, 43]. This finding suggests that different sites are specific to each of the grasp reflexes of the hands and feet within the nonprimary motor cortex. As demonstrated, each nonprimary motor area retains some degree of a somatotopically arranged map of the body that represents peripheral innervation, which is dominated by descending pathways from the area through connections to different levels of the spinal cord [54, 56–58]. We speculate that the grasp reflexes of the hands and feet may be elicited according to the specific location of each lesion corresponding to the fingers or toes on the map in nonprimary motor areas.

#### 2.3.3. Factors Affecting Responses in Infants

In normal circumstances, higher brain centers control the spinal mechanism that regulates the coordination among agonists, antagonists, and synergists in an adaptable manner by means of reciprocal innervation. In children with spastic type CP, however, deviation in terms of reciprocal innervation caused by damage to the pyramidal tract leads to excess cocontraction at proximal joints, whereas deviation leads to excess reciprocal inhibition through spastic antagonists at distal joints, inducing weakness of the agonists [62]. A diminished or negative plantar grasp reflex may be caused by weak toe flexors, as induced by excessive reciprocal inhibition of the flexors through the predominance of extensors over flexors in tonus in a lower limb in these children. They also exhibit the predominance of finger flexors over extensors in their upper limbs, which may cause a facilitated palmar grasp reflex on the affected side(s) in spastic hemiplegia and quadriplegia.

 On the other hand, in children with CP of the athetoid type, the deviation of reciprocal innervation always leads to excess reciprocal inhibition. Any attempt at movement produces excessive relaxation of the antagonists, inducing an extreme range of movements [62]. Although the released control of the spinal reflex mechanism because of a lesion in the basal ganglia seems to be the most likely cause of facilitation of the plantar grasp reflex in these children, the excessive relaxation of toe extensors, in response to the toe flexion at the moment of elicitation of the reflex, may be another factor causing an exaggerated response. Children with athetosis generally exhibit decreased tonus of finger muscles, especially of flexors, which causes a weak grasp and the release of objects too easily [62]. The relative predominance of finger extensors over flexors, in addition to the specific weakness of the finger muscles, may be a factor causing the diminished palmar grasp reflex in these children.

In infants, the maturation of cortical connections overrides the generators of primitive reflexes in the spinal cord and brain stem with age and eventually leads to the disappearance of the primitive reflexes and the emergence of righting and equilibrium reactions [34, 63]. The disappearance and emergence of these reflex mechanisms were demonstrated to be chronologically related to the attainment of motor milestones in normal children [24]. In children with MR, however, the process of evolution of these reflex mechanisms is prolonged, in accordance with the retardation of maturation in brain function, resulting in retention of primitive reflexes and the delayed attainment of motor milestones [24, 29, 34, 64].

## 3. The Moro Reflex

### 3.1. Elicitation

In his original method, Moro [9] elicited the reflex by hitting the pillow on either side of an infant's head with the hands. Later a variety of methods for eliciting the reflex were devised including hitting on the table surface, warm or cold application to the chest or stomach, and a tap on the abdomen [10, 11]. Nowadays, however, the head drop method is the most common, because a slight drop of the infant's head relative to the body axis in the supine position is generally accepted as the most effective technique for eliciting the reflex [10, 11, 19]. For elicitation in this method, the infant is held suspended in a symmetrical supine position with one of the examiner's hands behind the chest and the other supporting the head, and the head being held in a midline position and then dropped back a few cm. It is important to ensure that both the subject's hands are open at the moment of elicitation of the reflex so as not to provoke an asymmetrical response [14]. Infants should be tested while they are awake, but not crying [18].

 The drop of the baby method is an alternative one for eliciting the reflex: the infant is suspended horizontally, as in the head drop method, and then the examiner lowers his or her hands rapidly about 10 to 20 cm and brings them to an abrupt halt. There is no dorsiflexion of the neck with this technique [18]. On the other hand, Lesný [65] reported that a nociceptive stimulus applied to the infant's skin and subcutaneous tissue of the epigastrium by pinching was effective for eliciting the reflex.

 The initial phase of the response comprises abduction of the upper limbs at the shoulders and extension of the forearms at the elbows, with slight extension of the spine and retraction of the head. The forearms are supinated and the digits extended, except for the semiflexed index fingers and thumbs, forming the shape of a “C”. There is sometimes a slight tremor or clonus-like rhythmic movements of the limbs. Subsequently the arms adduct at the shoulders and the forearms flex at the elbows: the upper limbs describe an arc-like movement, bringing the hands in front of the body, which finally return to the original position [10, 11, 18]. The responses of the lower limbs are usually eliminated from the evaluation, because they show wide variability among the normal population [11, 19, 20]. With this reflex, habituation develops only on an experimental basis with intensively repetitive trials, that is, not in the clinical setting [17, 66]. No significant difference in the response has been reported at birth or through the first 5 months of age between the term cephalic-presenting and breech-presenting infant groups [67].

### 3.2. Clinical Significance

#### 3.2.1. Responses in Normal Infants

The study of the Moro reflex in normal term infants has been undertaken by many authors. The results obtained with the head drop method are well consistent. The reflex can be elicited in all infants during the first 12 weeks of age. After the neonatal period, however, the response becomes increasingly less typical with age, eventually consisting only of abduction and extension of the upper limbs. Beyond 12 weeks of age, the proportion of infants exhibiting a negative response rapidly increases, reaching about 80% at 20 weeks of age [25]. The reflex usually disappears by 6 months of age [11, 31, 68–70]. Several authors compared the Moro reflex in preterm infants tested at 40 weeks PCA or at 4 months of corrected age with that in term infants. Although none of the studies confirmed the outcome in the subjects, there is a general agreement as to the similarity in the response between the two groups, especially when only infants with no or low perinatal risk factors are compared [1, 71–75].

#### 3.2.2. Abnormal Responses

Based on the findings in normal infants, the absence or diminution of the Moro reflex within 2 to 3 months of age and the persistence of the response beyond 6 months of age can be regarded as abnormal. The absence of the response during the neonatal period and early infancy is of especial clinical significance and may indicate a compromised condition or disorder including birth injury, severe birth asphyxia, intracranial hemorrhage, infection, brain malformation, general muscular weakness of any cause, and CP of the spastic type [10, 31, 69, 76, 77]. On the other hand, a hyperactive response of the reflex is a common feature of neonatal withdrawal from maternal drug abuse including volatile substances, heroin, and opioids [78–80]. An exaggerated response may also be detected in infants with a severe bilateral intrauterine disturbance such as hydranencephaly [31].

 Asymmetry of the response is usually a sign of local injury. Damage to a peripheral nerve or cervical cord or a fracture of the clavicle may inhibit the reflex on the affected side. However, it should be noted that Dubowitz [14] demonstrated an asymmetrical response in normal infants. Their responses appeared to be related to the clenching of one fist during the procedure, and he considered that this might be caused by inhibition of the response on one side due to contraction of the finger flexors. Because Reiners et al. [81] found, in a prospective study, that none of 22 infants with a clavicular fracture exhibited an asymmetrical Moro response, the diagnostic value of the reflex for the detection of such fractures should not be overestimated. Retention of the reflex is common in children with MR without motor disturbance including Down's syndrome and in children with CP of the athetoid type [10, 69]. It is also sometimes observed in children with a severe brain malformation or with CP of the spastic type [31, 69].

### 3.3. Neural Mechanism

#### 3.3.1. The Reflex Center

Katona [17] reported that the Moro reflex could be elicited in anencephalic newborns with a nervous system that had developed only to the rostral level of the pons. In a study involving analysis of the relationship between the morphological structures of the rudimentary brain and primitive reflexes in six anencephalic newborns, Hanabusa [39] found that the Moro reflex could be elicited only when the vestibular nuclei were preserved. This finding indicates that the reflex is principally mediated by the vestibular nuclei. Rönnqvist et al. [82] reported that the average latency of the Moro reflex in 15 term neonates in the quiet awaking state detected with an optoelectronic device was 117.0 ms on the right arm and 129.2 ms on the left. These latencies are much longer than those of the spinal reflexes, clearly indicating that the reflex is mediated in the brain stem, not at the level of the spinal cord [83, 84]. Thus, the center of the Moro reflex seems to be in the lower region of the pons to the medulla.

#### 3.3.2. Afferent and Efferent Pathways

The origin of afferent pathways for the Moro reflex, whether it is primarily vestibular, proprioceptive, or exteroceptive, has been a main subject of discussion. The head drop, the most common way of eliciting the reflex, stimulates both the vestibular system and the proprioceptive receptors in the neck. Rönnqvist [13] investigated the reflex by tilting the table without extension of the infants' neck to eliminate the proprioceptive inputs from the cervical vertebrae and neck muscles. The response could be elicited in 225/250 trials (90%), and twenty-one of the 25 negative trials were made while the infants were sleeping or crying. Prechtl [12] also demonstrated that sudden raising or dropping of the infants, whose head, neck, and trunk were fixed in a plaster cast, could elicit the response. Bloomfield et al. [85] reported an infant with CHARGE syndrome who exhibited a persistent complete absence of the Moro reflex with preservation of other primitive reflexes. Moro's original method cannot yield a satisfactory response if the table is too stable to produce a change in position on striking and necessitates jolting movement of the table to elicit a response [10]. These findings support the view that this reflex is principally mediated by the vestibular system. In contrast to the grasp reflex, the Moro reflex has not been observed in a fetus, which is also in agreement with its vestibular origin, because fetuses are protected from acceleration or shaking in intrauterine life [68, 86].

On the other hand, Parmelee Jr. [11] found that vestibular stimulation was not sufficient for a good Moro response when neck movement was prevented. Prechtl [12] described an infant with bilateral absence of the inner ears who had exhibited a normal Moro response to a head drop, which was reported by Karlsson in an address to a study group in Oxford. These observations suggest that the proprioceptive inputs from the neck also contribute to elicitation of the reflex. There are direct and indirect, via the cervical cord, ascending pathways that originate in the proprioceptive receptors in the neck and connect with vestibular nuclei. These pathways originally send signals to the brain stem to regulate the neck righting [57]. The signals generated by the head drop may travel via these routes to reach and activate the reflex mechanism in the brain stem. The head drop method is most effective, because it produces a large number of ascending signals to the target through the two pathways and thereby induces a high level of neural excitation that acts on the reflex center.

 Although pinching of an infant's epigastrium has been reported to be effective for eliciting the reflex [65], a nociceptive stimulus is generally ineffective [10, 11]. Besides the primary somatosensory afferents, there is a path taken by nociceptor axons that reaches the pontine reticular formation, which has close interconnections with vestibular nuclei [87–89]. The nociceptive signals may travel via this pathway toward the reflex center in the brain stem. However, the level of neural excitation generated by nociceptive stimulation appears to be usually low, and the response can be elicited only when it infrequently exceeds the threshold of the reflex.

 The reflex center probably contains a number of interneurons, because of the relatively long latency. The routes of afferent pathways can be multiple, and the efferent pathways of the response seem to originate in the vestibulospinal and/or reticulospinal neurons, because the response can even be obtained in anencephalic newborns devoid of both corticospinal and rubrospinal neurons [17, 39]. Thus, the reflex movement is generated by the subcortical structures without cortical participation, which explains why focal cerebral injury does not cause distinct disturbance of the Moro reflex [31]. It is also noteworthy that no asymmetrical response is sometimes detected in neonates and young infants who later develop spastic hemiplegia. The primary motor cortex and nonprimary motor areas project a lot of neurons to different motor centers in the brain stem including vestibulospinal and reticulospinal neurons. The brain stem also receives inputs from the basal ganglia and cerebellum. The Moro reflex in infants disappears with age, due to the increased inhibition of these upper brain structures.

#### 3.3.3. The Moro Reflex and Startle Reaction

Although there has been much confusion regarding the Moro response and the startle reaction in the past, most authors agree today that they are different entities [10–12, 17]. The startle reaction, the response to a sudden stimulus, is one of the defensive reactions and consists essentially of flexion movements. It differs considerably from the Moro response primarily characterized by extension. Detailed observations with video recording [90] or film [91] and an electrophysiological study with surface electrodes [12] demonstrated the differences in motor behavior between them. Katona [17] found that the startle reaction induced by an auditory stimulus showed clear habituation in premature infants, whereas the Moro reflex did not, and that the startle reaction could not be elicited in anencephalic newborns, while the Moro reflex was always elicited in these infants. Pucher et al. [68] investigated the Moro reflex using a tilt table with simultaneous monitoring of autonomic parameters including respiration, heart rate, and transcutaneous pO2 and pCO2 and concluded that the reflex was not the result of a startle reaction, because of no significant alteration in these parameters.

## 4. Phylogenetic Meaning and Hierarchy of Responses

### 4.1. Phylogenetic Meaning

#### 4.1.1. The Grasp Reflex

In 1891, Robinson [92] described that a newborn human infant was able to support its own weight when it was holding on to a horizontal rod. He tested more than 60 infants under one month old and found that they were able to hang by their hands for at least 10 seconds, and one infant succeeded in hanging for 2 minutes and 35 seconds. Richter [46] had the opportunity of testing the palmar grasp reflex in five monkey infants. They showed a grasp reflex that was much stronger than that of human newborn infants. The time of hanging ranged from 7 to 33 minutes with only one hand. Brain and Curran [33] examined the plantar grasp reflex in nine adult anthropoid apes and monkeys and found that none of the monkeys showed the grasp reflex and that the prehensile function of the adult monkey's feet was highly specialized, responding to a variety of stimuli. They also observed a number of infant monkeys, some within a few days of birth, slung beneath the mother's belly or clinging to her flanks only with their hands and feet, receiving no support from her. Although only a day or two old, they held on while she jumped from bough to bough and did not leave her until they were weaned.

 Based on these findings, the grasp reflex of the hands and feet in human infants could be regarded as a rudiment of phylogenetic functions that were once essential for monkey infants in arboreal life and that have lost their usefulness in the human species [28, 33, 62]. As McGraw [93] stated, modern infants, as well as their fairly recent human antecedents, do not need to hang on with their hands and feet from the moment of birth.

#### 4.1.2. The Moro Reflex

In his original paper, Moro [9] emphasized the clasping aspect of the reflex and claimed that the reflex is a primitive movement analogous to that in young apes and bats, by which they instinctively embrace or cling to their mothers. However, the question has been raised as to his biological interpretation by several authors. Parmelee Jr. [11] stated that Moro's emphasis in his belief that the reflex is an atavistic lifesaving reflex resulted in some of the present-day confusion. Mitchell [10] described that in the Moro response, the essential component is the extension movement, and thus it should not be called an “embrace” or “clasp” reflex. On the other hand, Amiel-Tison and Grenier [94] commented that the Moro reflex can be seen as a hindrance to voluntary motor activity, and this parasitical movement is a nuisance that the newborn retains until a subsequent stage of maturation when inhibitory brain function begins. The unique movement behavior of the Moro reflex is thus difficult to understand, and its phylogenetic meaning remains unclear.

### 4.2. Hierarchy of the Reflex Responses

Several authors observed in human newborns that the palmar grasp reflex inhibits the Moro reflex [12, 15, 16, 22, 91]. Later, Katona [17] found in newborn apes and monkeys that the Moro reflex was regularly observed and it was inhibited on elicitation of the palmar grasp reflex. Because this interrelation between the grasp reflex and the Moro reflex would be essential for newborn animals to keep clinging and to prevent a fall, the interrelation observed in human newborns could also be regarded as a phylogenetic rudiment. Katona [17] noted another fact that a sudden auditory stimulus triggered a strong reinforcement of the grasp reflex in such young animals, which also supports the view that the startle reaction is one of the defensive reactions. Pollack [22] found, in term human newborns, that sucking increased the palmar grasp reflex, whereas head rotation had no influence on this grasp reflex. Lippmann noted in human newborns that the Babkin reflex consisting of head flexion and mouth opening in response to pressure on the palms of both hands was not obtained during sucking [95]. Brown and Fredrickson [15] also observed in human newborns that the palmar grasp reflex did not affect the sucking reflex but that the sucking reflex increased the strength and the duration of the palmar grasp reflex. If the same interrelation between the sucking reflex and the palmar grasp reflex exists in monkey infants, it would be very helpful for them to firmly cling to their mother while feeding. The asymmetrical tonic neck reflex (ATNR) elicited by head rotation usually induces extension of the upper and lower limbs on the face side in infants. If the palmar grasp reflex is predominant over the ATNR in monkey infants, as demonstrated in human infants, head rotation would not bring any reduction in the response of the grasp reflex, and the animal infant would keep clinging to its mother. Since the Babkin reflex is regarded as the rudiment of hand-mouth coordination for preying in animals [96, 97], it appears to be rational that the reflex is unable to be elicited during feeding. Thus, the hierarchical interrelations among the primitive reflexes observed in human newborns seem to be indispensable for monkey newborns for their essential behavior such as feeding, moving, and preventing a fall, although the interrelations have not been fully elucidated in monkey infants.

Based on the findings already mentioned in this paper, the Moro reflex in the young monkey would be elicited when the vestibular system is stimulated with abrupt tilting of the body or head while it is being passively held by its mother without active clinging caused by the palmar grasp reflex. In this situation, the mother would notice her baby was off balance from its exaggerated reflex movement, and she would immediately try to seize the baby to prevent a fall. It might be possible to assume that the Moro reflex in monkey neonates plays a role in such interaction between mother and child for protection against a fall. To clarify the meaning of the Moro reflex, it appears necessary to determine in monkeys in what situation the reflex is elicited and how the response works in the mother and child.

## 5. Conclusions

The palmar grasp reflex in infants has diagnostic significance. The absence or a weak response of the reflex during early infancy may reflect peripheral nerve or spinal cord involvement or may predict the development of athetoid type CP, whereas the response may be hyperactive in children with spasticity in their upper limbs. The plantar grasp reflex is also of high clinical significance, especially in terms of the detection of spasticity. No reflex, or a diminished one, during early infancy is often a sensitive predictor of the development of spastic CP. The grasp reflex of the hands and feet is mediated by the spinal reflex mechanism, which, however, appears to be under regulatory control of nonprimary motor areas through the spinal interneurons. The absence of the Moro reflex during the neonatal period and early infancy is highly diagnostic, indicating a variety of compromised conditions. The center of the reflex is probably in the lower region of the pons to the medulla. The grasp reflex of the hands and feet in human infants could be regarded as a rudiment of phylogenetic function, whereas the phylogenetic meaning of the Moro reflex remains unclear. The hierarchical interrelations among the primitive reflexes seem to be essential for monkey newborns for their arboreal life, although it has not been fully elucidated. The possible role of the Moro reflex in these newborns was discussed in relation to the interrelations.
